# Addition of quantitative MRI to the routine clinical care of patients with multiple sclerosis—Results from the MAGNON project

**DOI:** 10.1002/brb3.3548

**Published:** 2024-06-06

**Authors:** Verena Isabell Leussink, Manda Jankovic, Marie Groth, Katrin Schuh, Inessa Schwab Sauerbeck, Olaf Hoffmann

**Affiliations:** ^1^ Praxis Neurologie in Meerbusch Meerbusch Germany; ^2^ Sauerlandklinik Hachen Sundern Germany; ^3^ Clinical Research Neuroscience Novartis Pharma GmbH Nuremberg Germany; ^4^ St. Josefs‐Krankenhaus Potsdam‐Sanssouci Potsdam Germany; ^5^ Medizinische Hochschule Brandenburg Theodor Fontane Neuruppin Germany

**Keywords:** brain volume, multiple sclerosis phenotype, quantitative magnetic resonance imaging, secondary progressive multiple sclerosis, thalamic volume

## Abstract

**Background:**

The revised Lublin classification offers a framework for categorizing multiple sclerosis (MS) according to the clinical course and imaging results. Diagnosis of secondary progressive MS (SPMS) is often delayed by a period of uncertainty. Several quantitative magnetic resonance imaging (qMRI) markers are associated with progressive disease states, but they are not usually available in clinical practice.

**Methods:**

The MAGNON project enrolled 629 patients (early relapsing‐remitting MS (RRMS), *n* = 51; RRMS with suspected SPMS, *n* = 386; SPMS, *n* = 192) at 55 centers in Germany. Routine magnetic resonance imaging (MRI) scans at baseline and after 12 months were analyzed using a centralized automatic processing pipeline to quantify lesions and normalized brain and thalamic volume. Clinical measures included relapse activity, disability, and MS phenotyping. Neurologists completed questionnaires before and after receiving the qMRI reports.

**Results:**

According to the physicians’ reports, qMRI results changed their assessment of the patient in 31.8% (baseline scan) and 27.6% (follow‐up scan). For ∼50% of patients with RRMS with suspected SPMS, reports provided additional information that the patient was transitioning to SPMS. In >25% of all patients, this information influenced the physicians’ assessment of the patient's current phenotype. However, actual changes of treatment were reported only in a minority of these patients.

**Conclusions:**

The MAGNON results suggest that standardized qMRI reports may be integrated into the routine clinical care of MS patients and support the application of the Lublin classification as well as treatment decisions. The highest impact was reported in patients with suspected SPMS, indicating a potential to reduce diagnostic uncertainty.

## Graphical abstract

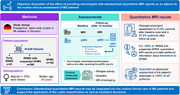

## 1 INTRODUCTION

Multiple sclerosis (MS) is a chronic demyelinating, autoimmune‐inflammatory, and neurogenerative disease affecting the brain and spinal cord, (Dobson & Giovannoni, 2019) It is estimated that there are 2.9 million people worldwide living with MS. With the prevalence increasing globally, more than 1 in 500 population may be affected in parts of Europe and northern America(MS International Federation, 2023). MS is a leading cause of nontraumatic disability in young adults (Dobson & Giovannoni, 2019).

Based on the clinical course, a distinction is made among different forms of MS. Initially, approximately 85% of patients present with relapsing‐remitting MS (RRMS). Up to 90% of untreated RRMS patients transition to secondary progressive MS (SPMS) when followed up for 25 years or longer (Rice, 2002). SPMS is characterized by progressive deterioration of disability, independent of relapses. In recent SPMS clinical trials, most participants had been diagnosed within 10 years from RRMS onset (Ziemssen et al., 2023). However, large variations are observed, and transition is probably delayed by disease‐modifying therapy (Brown et al., 2019). Up to 15% of MS patients experience a progressive course from disease onset, referred to as primary progressive MS (PPMS) (Miller & Leary, 2007).

A framework for classifying the phenotype of MS was originally presented in 1996 and revised in 2013. Referenced herein as Lublin classification, there is a categorical distinction between relapsing versus progressive MS, supplemented by the presence or absence of disease activity, and of progression of disability (Lublin et al., 2014). Disease activity may occur as a clinical relapse, or by the emergence of contrast‐enhancing or new or enlarging T2 lesions (NET2Ls) on magnetic resonance imaging (MRI). In addition to clinical assessment of relapses and progression, an annual MRI is considered useful, at least in RRMS patients, to detect subclinical disease activity (Wattjes et al., 2021).

This categorization of MS according to phenotype, disease activity, and progression is reflected in therapeutic strategies and in the specific indications of presently available disease‐modifying treatment (DMT) options. Primarily addressing relapses and the formation of new lesions, most DMTs are licensed for treating relapsing MS, whereas efficacy in progressive MS has been proven in dedicated clinical trials only for siponimod (SPMS) and ocrelizumab (PPMS) (Kappos et al., 2018; Montalban et al., 2017). Siponimod shows superior efficacy in patients still presenting with superimposed relapses or MRI activity, so‐called active SPMS.

Timely recognition of the transition to SPMS may enable earlier use of approved pharmacological and nonpharmacological therapy, which potentially could translate into better outcomes (Ziemssen et al., 2023). Mechanisms that contribute to the relapse‐independent progression of disability and the transition to SPMS are active also during the RRMS phase (Giovannoni et al., 2022; Kuhlmann et al., 2023; Lublin et al., 2022), thereby challenging physicians to recognize the transition phase (Ziemssen et al., 2020). Based on clinical judgment, the diagnosis of SPMS is usually made retrospectively, and with several years of delay after the onset of the first clinical signs of progression (Katz Sand et al., 2014; Kremenchutzky et al., 2006). There are no generally accepted diagnostic criteria, owing to a large heterogeneity in clinical manifestation and the absence of routinely available biomarkers (Wattjes et al., 2021). Several MRI findings are linked to progressive disease states, including accelerated whole brain and grey matter volume loss. So far, quantitative MRI (qMRI) methods and assessment of brain and spinal cord atrophy are, however, not recommended for diagnostic or routine monitoring purposes (Sastre‐Garriga et al., 2020; Wattjes et al., 2021).

The MAGNON project aims to evaluate the effect of providing neurologists with standardized qMRI reports as an adjunct to the routine clinical assessment of MS patients. We focus on the perspective of the treating physicians regarding additionally gained information, its impact on the categorization of patients according to the modified Lublin classification, and therapeutic consequences.

## 2 METHODS

### 2.1 Study design and patient population

MAGNON was a prospective nationwide project conducted between March 1, 2020, and February 18, 2023, in Germany, where data were collected from office‐based neurologists, hospitals, and rehabilitation centers. Adult (≥ 18 years old) male or female patients diagnosed with early RRMS, RRMS with suspected transition to SPMS, or SPMS who provided their signed informed consent were included, whereas those enrolled in other non‐interventional studies or clinical trials were excluded. Enrolled patients who did not undergo a baseline MRI examination were also excluded from the study.

#### 2.1.1 MRI data acquisition and reporting

At project initiation, a questionnaire was sent to all physicians regarding the overall MS patient population at each participating site. For each participating patient, two MRI scans including 3D T1‐weighted gradient echo and 2D/3D FLAIR sequences were performed in the clinical routine setting, including a baseline scan and a follow‐up scan after up to 12 months. In addition to routine evaluation and reporting by the local radiologist, a remote qMRI analysis was performed using a standardized processing pipeline (Biometrica MS, jung diagnostics GmbH) (Opfer et al., 2016; Spies et al., 2013). Segmentations of lesions and tissue components as well as atlas‐based volumetry are based on the Statistical Parametric Mapping Tool (https://www.fil.ion.ucl.ac.uk/spm/). Application to MS has previously been reported, including an extensive bibliography of validation studies (Raji et al., 2018). Biometrica MS has been licensed for clinical use under the “Conformité Européenne” mark. Quantitative reports were provided to the neurologists, including T2 lesion number and volume, and normalized brain and thalamic volumes. For the follow‐up MRI, changes in brain and thalamic volume relative to the baseline scan were reported. Volumetric data were obtained using the optimized SIENA pipeline and reported as *z*‐scores, indicating the difference in standard deviations (SDs) from the mean value of a reference cohort matched for age and head size.

Before each MRI and after receiving the quantitative report, the treating neurologists filled in questionnaires on the patient's disease activity, disability according to the Expanded Disability Status Scale (EDSS) (Kurtzke, 1983), and MS phenotype (Lublin et al., 2014). Here, RRMS patients were categorized into those with active (relapse and/or MRI activity) versus not active disease. Considering a deterioration of clinical disability over the last year, SPMS patients were categorized into four subgroups, that is, active with progression, active without progression, not active with progression, and not active without progression (stable condition). Physicians also reported on the perceived value of the quantitative report and its impact on their classification of the patient but were not expected to perform independent validation of the quantitative results.

### 2.2 Outcomes

Outcome measures included assessment of disease activity (relapses), EDSS score, and MS phenotype. On MRI, T2 lesion count, T2 lesion volume, normalized brain, and thalamic volumes were quantified at baseline and at follow‐up. The use of gadolinium was facultative in this project. Additional outcomes included changes in clinical presentation and MS treatment over the 12‐month period, and the impact of MRI results from the physicians’ perspective.

### 2.3 Statistical analysis

Data collected through questionnaires were recorded in electronic case report forms and are presented descriptively. Only signed records were included in the analyses. For patients with only partially signed visits, the chronological order of the visits as well as the continuity of the visits were considered. As patients could be recategorized during follow‐up, descriptive statistics are reported for all available scans per category at each time point. In contrast, NET2Ls were determined for matched scans, considering the final clinical categorization. Categorical variables were represented as frequencies (percentages), and continuous variables were represented as mean (SD) or median (interquartile range). Analysis was performed using SAS version 9.4.

### 2.4 Ethical standards

The study protocol, data handling, and patient materials were approved by the responsible ethics committee (Brandenburg State Medical Association, Cottbus, Germany; approval number S11(bB)/2020). All study procedures fully conformed to the Declaration of Helsinki.

### 2.5 Data sharing and accessibility

The data that support the findings of this study are on file at Novartis Pharma GmbH and Novartis Pharma AG. Due to licensing restrictions, they are not publicly available. Data are, however, available from Inessa Schwab Sauerbeck (inessa.schwab_sauerbeck@novartis.com) upon reasonable request and may only be used with permission of Novartis Pharma GmbH and Novartis Pharma AG.

## 3 RESULTS

### 3.1 Patient population across all study sites

**FIGURE 1:**
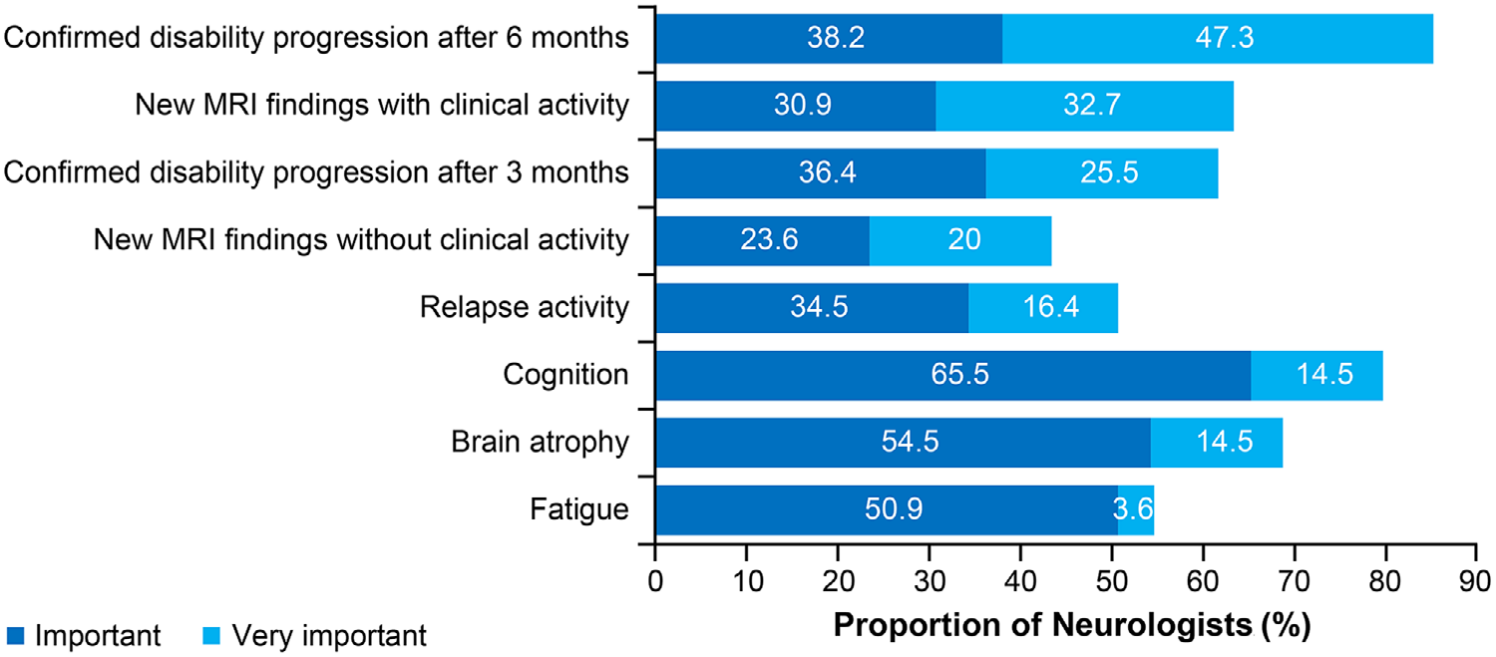
Rating of factors used to evaluate phenotype transition from relapsing‐remitting multiple sclerosis (RRMS) to secondary progressive multiple sclerosis (SPMS). MRI, magnetic resonance imaging.

MAGNON involved 55 sites, which together had an estimated 21,266 MS patients registered at the time of project initiation. Most patients (61.8%) were classified as MS with a predominantly relapsing course. Of the patients with RRMS, a mean of 20.6% was considered in transition to SPMS. The most important attributes (rated as “very important”) in the physician's decision to diagnose transition to SPMS are 3‐month/6‐month confirmed disability progression (CDP) and new MRI findings with and without clinical activity, whereas cognition, brain atrophy, and fatigue were rated as “important” (Figure 1).

### 3.2 Patient population included in the analysis

Of 691 enrolled patients, 62 were excluded owing to missing signature (*n* = 12) or missing the first MRI (*n* = 50). Thus, a total of 629 MS patients are included in the present analysis. Of these, 8.1% had early RRMS (*n* = 51), 61.4% had RRMS with suspected transition to SPMS (*n* = 386), and 30.5% had SPMS (*n* = 192); the majority of patients were female (Table 1). The median age of the study population was 50.0 years and the median time since MS diagnosis was 12 years. Time since diagnosis was censored in five patients due to obvious error (calculated age at diagnosis <3 years). On average, enrolled RRMS patients with suspected SPMS were about 10 years older than patients with early RRMS (47.6 years vs. 35.2 years) and younger than patients diagnosed with SPMS (55.9 years). In patients with RRMS with suspected SPMS, time since first suspecting SPMS ranged between 0 and 10 years, with a mean of 1.5 years.

**TABLE 1:** Demographics and baseline characteristics.

Characteristic^a^	Early RRMS (*N* = 51)	RRMS with suspected SPMS (*N* = 386)	SPMS (*N* = 192)
**Age, years**	34 [19–67]	48 [20–75]	56 [33–77]
**Female, *n* (%)**	39 [76.5]	268 [69.4]	135 [70.3]
**Time since diagnosis, years**	1 [0–3]	12 [0–47]	17.5 [1–39]
**EDSS (mean [SD])**	1.4 [1]	3.0 [1.7]	5 [1.5]
**Time since last relapse, years (mean [SD])**	1.25 [1.25]	3.5 [3.31]	5.1 [5.48]

Abbreviations: EDSS, Expanded Disability Status Scale; RRMS, relapsing‐remitting multiple sclerosis; SD, standard deviation; SPMS, secondary progressive multiple sclerosis.

^a^ If not indicated otherwise, data are presented as median [IQR].

### 3.3 MS phenotyping

#### 3.3.1 MS phenotyping in SPMS patients

**FIGURE 2:**
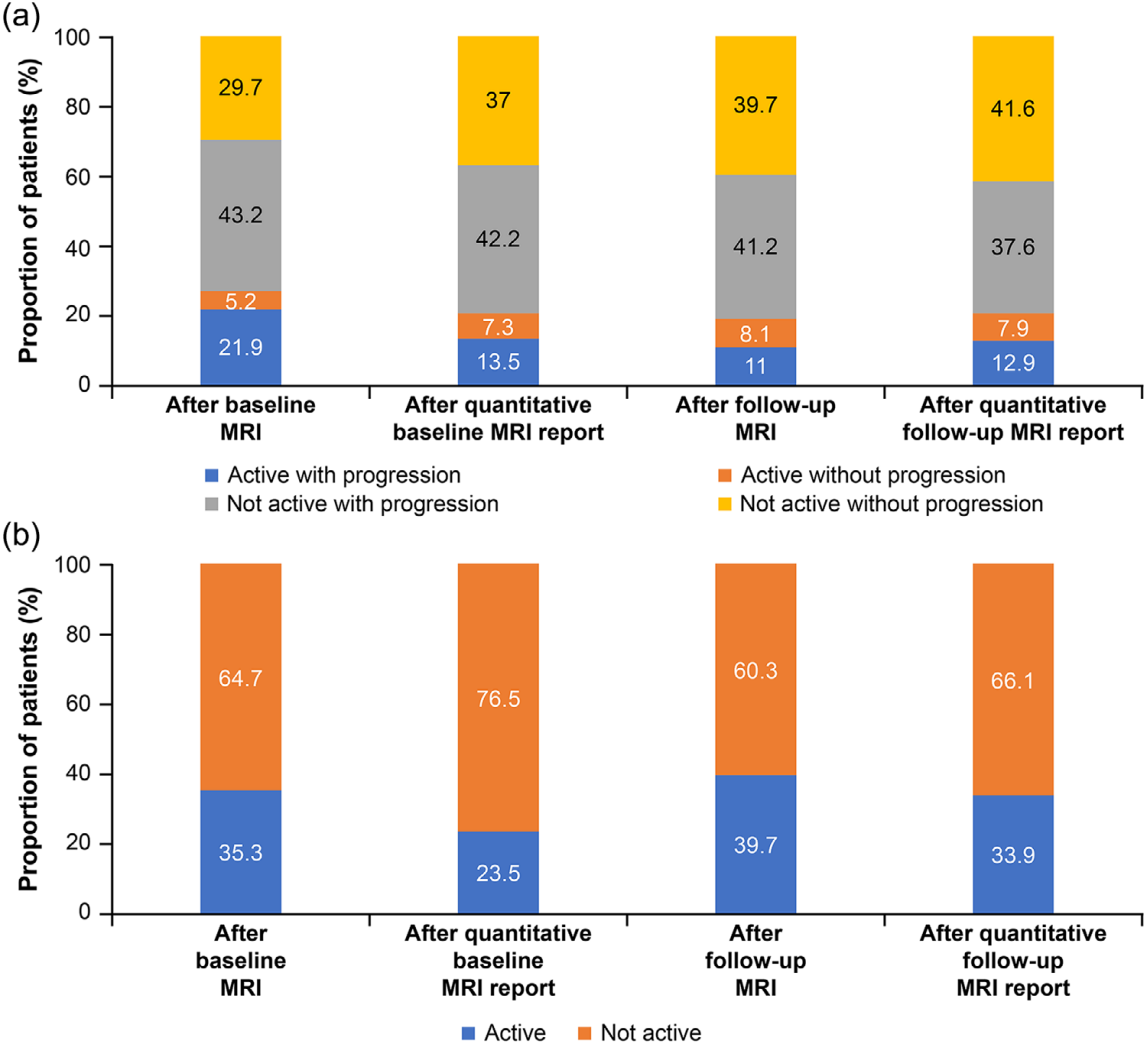
Phenotype in patients with (a) secondary progressive multiple sclerosis (SPMS) and (b) early relapsing‐remitting multiple sclerosis (RRMS) at quantitative baseline and follow‐up magnetic resonance imaging (MRI).

Considering clinical and routine MRI findings, most patients with SPMS were categorized at baseline as “not active with progression” (43.2%), followed by “not active without progression (stable)” (29.7%), “active with progression” (21.9%), and “active without progression” (5.2%) (Figure 2a). After including the baseline qMRI report, classification was “not active with progression” in 42.2%, followed by “not active without progression (stable)” (37.0%), “active with progression” (13.5%), and “active without progression” (7.3%).

After the follow‐up MRI, SPMS patients were categorized as “not active with progression” (41.2%), followed by “not active without progression (stable)” (39.7%), “active with progression” (11.0%), and “active without progression” (8.1%). Similar results were reported after receiving the follow‐up qMRI report.

#### 3.3.2 MS phenotyping in early RRMS patients

At baseline MRI, 64.7% of patients were classified as not active and 35.3% as active. Following the baseline qMRI report, the proportion of patients classified as not active increased to 76.5%, whereas the proportion of patients classified as active decreased to 23.5%.

After the follow‐up MRI, 60.3% of patients were classified as not active and 39.7% as active (Figure 2b). Again, the inclusion of the qMRI report increased the proportion of patients classified as not active to 66.1%, whereas those classified as active decreased to 33.9%.

### 3.4 Quantitative MRI analysis

#### 3.4.1 T2 lesions and volume

qMRI reports were successfully generated for all studies that were sent for central evaluation. Patients diagnosed with SPMS exhibited the highest number and volume of lesions, whereas those with early RRMS had the lowest values at baseline and follow‐up MRI.

**FIGURE 3:**
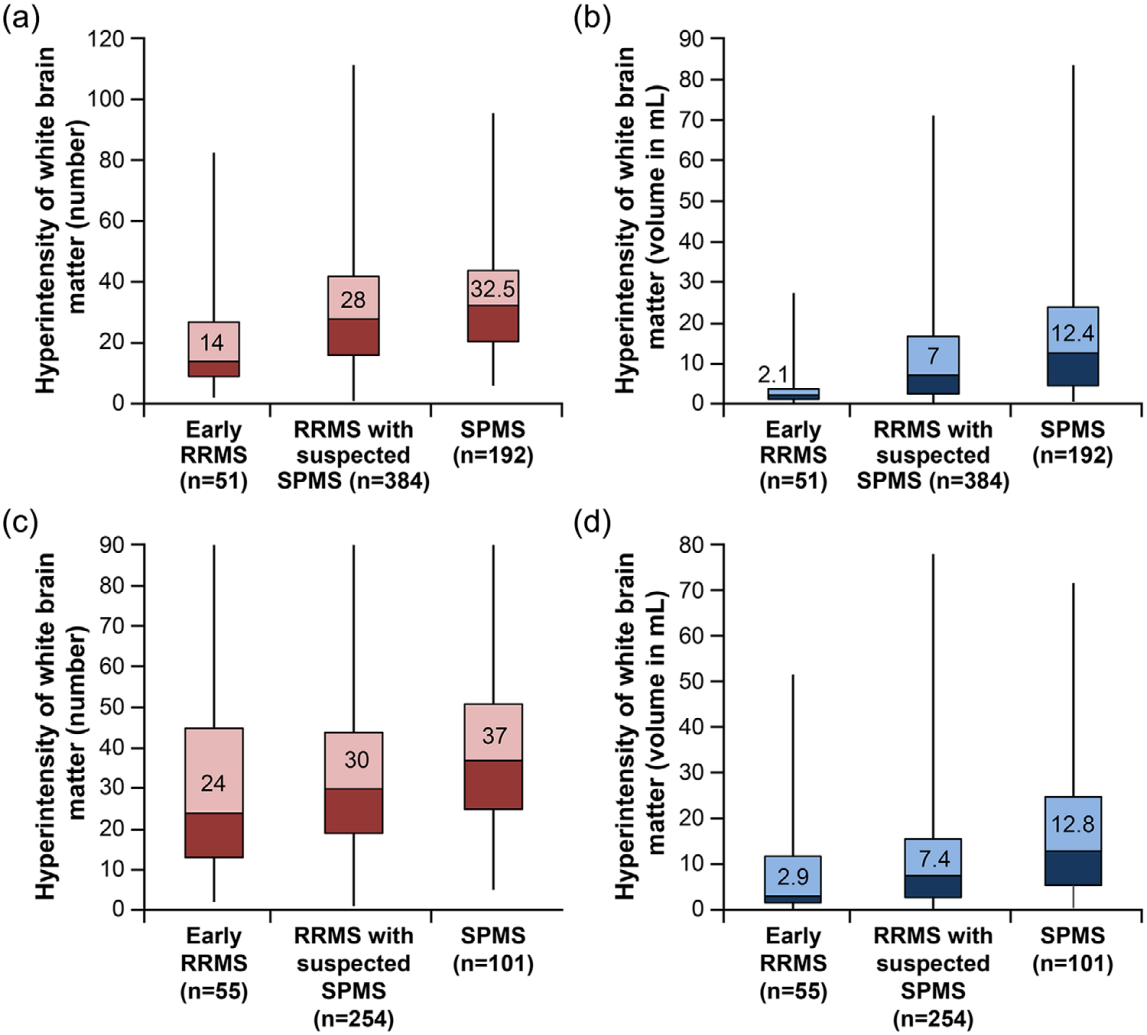
White matter lesion number (a and c) and volume (b and d) by distinct subgroups at baseline (a and b) and follow‐up MRI (c and d). MRI, magnetic resonance imaging; RRMS, relapsing‐remitting multiple sclerosis; SPMS, secondary progressive multiple sclerosis.

At baseline MRI, qMRI analyses of T2 hyperintense white matter lesions revealed a median number of 28.0 per patient (mean [SD]: 31.6 [19.5]; *N* = 627) and a median volume of 7.3 mL (mean [SD]: 12.9 [14.4] mL) across all subgroups (Figure 3a,b **)**. The median number of Gd+ T1 lesions was 0.0 in the overall population (data available from 83 patients) and in each subgroup (early RRMS, RRMS with suspected SPMS, SPMS). At follow‐up, the number of T2 hyperintense lesions per patient had increased to 32.5 (mean [SD]: 35.5 [21.0]; *N* = 410), whereas the median lesion volume was 7.9 mL (mean [SD]: 12.9 [14.1] mL) (Figure 3c,d). Again, the median number of Gd+ T1 lesions was 0.0 in the overall population (*N* = 60) and in each subgroup. The median number of NET2Ls at follow‐up was 0 in each subgroup. The mean number [SD] of NET2L was 1.1 [2.9] in patients with early RRMS (*N* = 48), 1.0 [2.8] in patients with suspected transition to SPMS (*N* = 218), and 0.6 [1.7] in patients with a diagnosis of SPMS (*N* = 85).

#### 3.4.2 Normalized brain and thalamic volume

**FIGURE 4:**
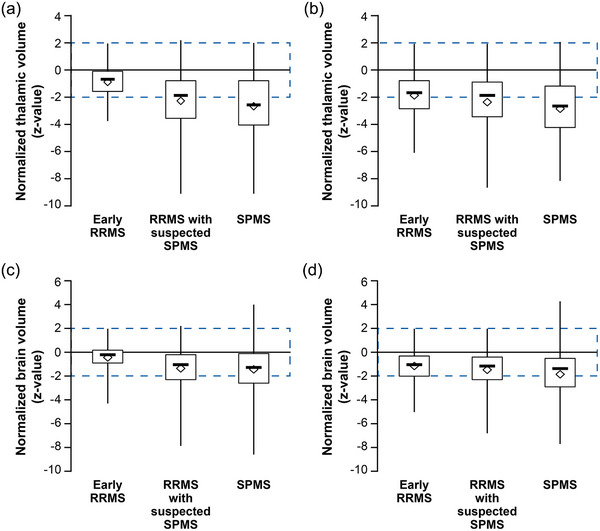
Normalized thalamic volume according to magnetic resonance imaging (MRI) reports at (a) baseline and (b) follow‐up; normalized brain volume according to MRI reports at (c) baseline and (d) follow‐up. The box plot shows median (solid line), mean (diamond shape). Normalized brain and thalamic volumes (*z*‐values) are adjusted for age and head size and show the deviation from the mean of a reference population of healthy individuals in units of the standard deviation. Light blue areas represent 95% of all healthy individuals (mean ± 1.96 standard deviations). Values below the cut‐off of −1.96 represent an abnormal brain/thalamic volume reduction with a maximal error probability of 2.5%. (a and c): early RRMS: *N* = 50, RRMS with suspected SPMS: *N* = 366, SPMS: *N* = 183. (b and d): early RRMS: *N* = 55, RRMS with suspected SPMS: *N* = 245, SPMS: *N* = 94. RRMS, relapsing‐remitting multiple sclerosis; SPMS, secondary progressive multiple sclerosis.

At baseline MRI, normalized thalamic and whole brain volumes across all groups showed a median *z*‐value of −1.9 (mean [SD]: −2.3 [2.2]; *N* = 599) and −1.1 (mean [SD]: −1.3 [1.7]; *N* = 599), respectively. At follow‐up MRI, the median *z*‐values were −2.1 (mean [SD]: −2.4 [2.1]; *N* = 394) and −1.2 (mean [SD]: −1.4 [1.6]; *N* = 394). At baseline MRI, the largest deviations in the normalized thalamic (−2.5 mL) and brain volumes (−1.3 mL) were observed for patients with a current diagnosis of SPMS, whereas the smallest deviations were found in the group with early RRMS (*z*‐value for thalamic volume: −.7, brain volume: −.4). Similar results were observed at the follow‐up MRI (Figure 4).

### 3.5 Changes in clinical presentation over 12 months

**FIGURE 5:**
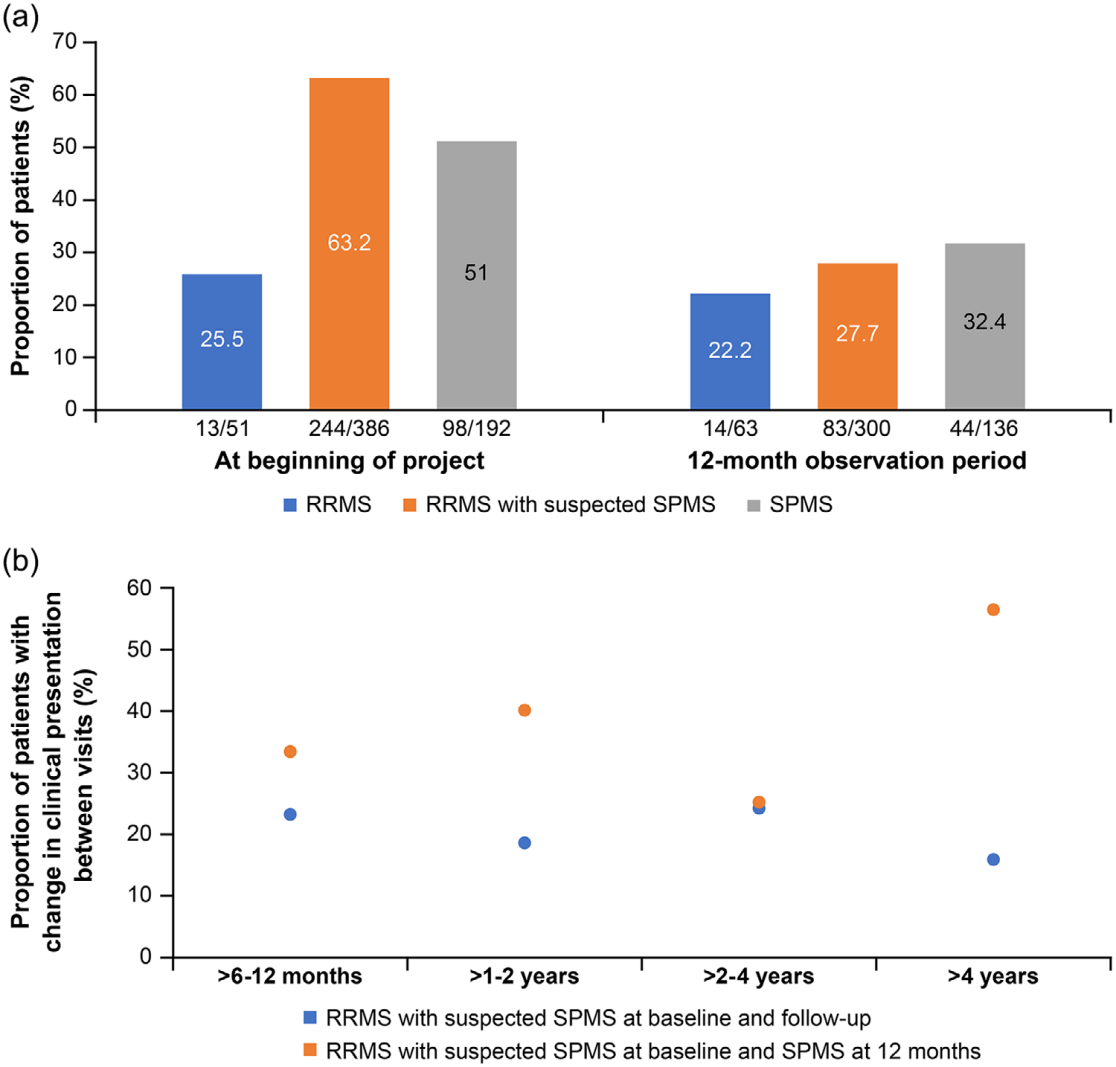
(a) Proportion of patients with worsening in clinical presentation according to multiple sclerosis (MS) phenotype at baseline and after 12 months and (b) relapsing‐remitting MS (RRMS) patients with suspected secondary progressive MS (SPMS) at baseline and SPMS diagnosis at 12 months: duration of therapy to date stratified by changes in clinical presentation. n, number of patients who are at corresponding category; N, total number of patients.

At the baseline MRI, changes in clinical presentation since initiation of the current therapy were reported in the majority of patients with suspected transition to SPMS or a current diagnosis of SPMS (Figure 5a). During the 12‐month observation period, changes in presentation were less frequent and more evenly distributed between groups. At both time points, increased fatigue, disability progression, or deterioration of cognition were the most common concerns.

When categorized as RRMS with suspected SPMS at baseline, a change in clinical presentation between visits was more frequent in patients who were reclassified to SPMS at follow‐up than in those who remained classified as RRMS with suspected SPMS (Figure 5b). The difference appeared to increase with a longer duration of therapy.

### 3.6 Therapy at baseline and changes over 12 months

At the initial MRI, 80.8% (312/386) of RRMS with suspected SPMS and 64.1% (123/192) of SPMS patients were on DMTs. Among the early RRMS patients, 25.5% (13/51) had changed their therapy at least once (and up to three times) prior to enrolment. At the time of follow‐up, 23.8% (15/63) of patients with early RRMS and 16.7% (50/300) of patients with RRMS with suspected SPMS had had a change in therapy. Among SPMS patients who received DMTs at baseline, 79.1% (53/67) continued treatment, whereas 29.3% (12/41) of those not treated at baseline had initiated therapy. Follow‐up data on DMT use were missing for 48 patients with early RRMS and 200 patients with RRMS with suspected SPMS.

### 3.7 Impact of quantitative MRI results from the physicians’ perspective

**FIGURE 6:**
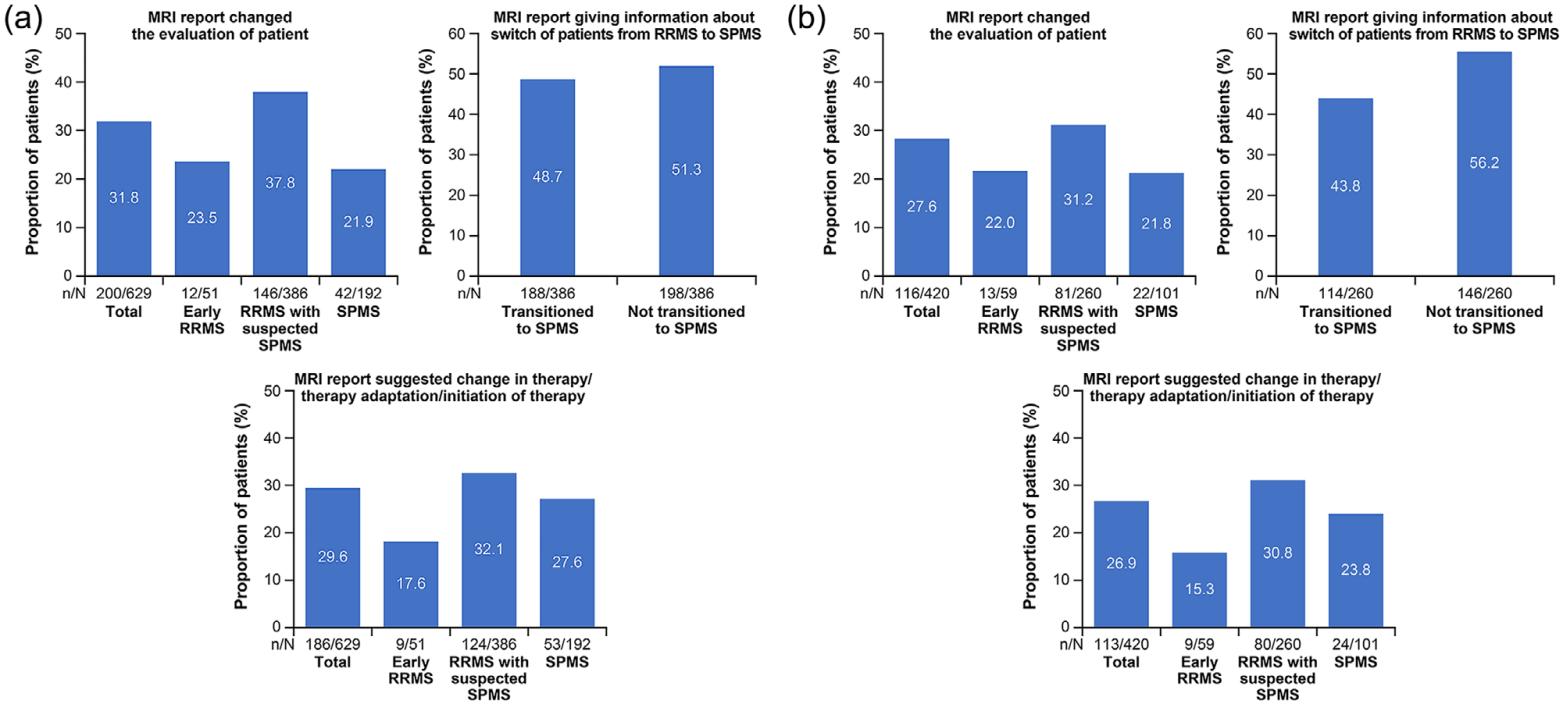
Impact of magnetic resonance imaging (MRI) results on evaluation of patient at baseline (a) and follow‐up MRI (b). RRMS, relapsing‐remitting multiple sclerosis; SPMS, secondary progressive multiple sclerosis; n, number of patients in the corresponding category; N, total number of patients.

The additional information from the qMRI reports had an impact on the physicians’ assessment of the current patient phenotype in more than 25% of patients; specifically, 31.8% after the baseline MRI and 27.6% after the follow‐up MRI. For RRMS patients with suspected SPMS, the quantitative report provided additional information indicating that 48.7% of patients with baseline MRI and 43.8% with follow‐up MRI were in transition from RRMS to SPMS (Figure 6). Furthermore, qMRI reports were considered to indicate a change in therapy for 32.1% and 30.8% of RRMS patients with suspected SPMS after the baseline and follow‐up MRI, respectively.

## 4 DISCUSSION

Using the task of categorizing the MS phenotype as a clinically relevant example, the MAGNON project offers insight on how the availability of qMRI results might affect and improve the routine care of MS patients. With the participation of 55 sites that provide continuous care to more than 21,000 MS patients (about 8% of all MS patients in Germany), this pilot study is representative of the current treatment landscape in Germany and beyond.

Regarding their usual diagnostic approach (not including qMRI), physicians reported relying on a wide range of factors when deciding whether a patient was transitioning to SPMS. Disease activity, including relapses and MRI findings, as well as CDP were regarded as important or very important, indicating strong consensus with the modified Lublin classification. In addition to physical disability, most physicians viewed neuropsychological impairment, including cognitive function and fatigue, as important or very important. In clinical routine, cognitive testing is, however, not usually performed or limited to screening instruments such as the Symbol Digit Modalities Test, providing insufficient assessment of cognitive functions (Leach et al., 2022). Although fatigue is a common concern of MS patients, it is multifactorial in origin and cannot be measured in an objective manner (Zimek et al., 2023). In MAGNON, observation for changes in the clinical presentation appeared to offer limited information during 12 months of follow‐up. Instead, differences between disease categories became apparent in retrospect and after longer treatment duration, as has been reported (Katz Sand et al., 2014).

In contrast to the usual diagnostic approach detailed above, accelerated atrophy of the whole brain and subcortical grey matter can consistently be demonstrated in SPMS patients and predicts further disability progression (Eshaghi et al., 2018; Hänninen et al., 2020; Magon et al., 2020; Radue et al., 2015; Rocca et al., 2021). Important caveats regarding the use of atrophy measures in the routine care of MS patients have been pointed out, including physiological and technical confounders as well as DMT effects (Sastre‐Garriga et al., 2020). As the most important practical barrier to implementation, volumetry and segmentation of lesions are not presently applied to routine MRI registrations.

In MAGNON, qMRI reports were provided to the treating neurologists for enrolled patients. Considering the quantitative results, patients with SPMS, as expected, had the highest number and volume of white matter lesions both on the baseline and on follow‐up MRI studies. This was followed by RRMS patients with suspected SPMS, whereas patients with early RRMS had the lowest values. Considering the increase in T2 lesion number on follow‐up, inflammatory disease activity was highest in the early RRMS group, whereas lesion dynamics are known to decrease with longer disease duration (Li et al., 2006; Uher et al., 2021).

In the outpatient setting, MRI studies are frequently performed by general radiologists who are not specialized in the evaluation of MS patients. Especially in individuals with a high lesion load, the quantification of NET2Ls may be challenging on visual interpretation alone, as is the determination of changes in lesion volume (Wattjes et al., 2021). Consequently, information with relevance to the categorization as active versus not active disease may be inaccurate or missing (Kappos et al., 2018).

Whole brain atrophy and atrophy of the cortical and subcortical grey matter are increasingly considered an important prognostic marker in MS patients. Even after the first relapse, reduced whole brain and grey matter volumes predict disability progression (Pérez‐Miralles et al., 2013; Popescu et al.,^2013^). Neurodegeneration and progression of disability are particularly linked to thalamic atrophy (Azevedo et al., 2018; Eshaghi et al., 2018). Compared to early RRMS, SPMS patients show more rapid grey matter atrophy (Eshaghi et al., 2018; Rocca et al., 2021).

In the present study, mean normalized thalamic and brain volumes were reduced across all patient groups when compared to the mean values of a reference population (Hänninen et al., 2020; Radue et al., 2015). Almost one in two RRMS patients with clinically suspected transition to SPMS showed an abnormally reduced thalamic volume. The *z*‐scores in this group were markedly heterogeneous, reflecting the difficulties in defining and recognizing these patients on clinical grounds alone. If considered a surrogate marker of disability progression, the inclusion of thalamic atrophy could add to the stratification of patients suspected of transitioning to SPMS.

At the core of this investigation, physicians provided feedback on the usefulness and impact of the qMRI reports. According to the questionnaires answered after the qMRI reports, the reports changed the neurologist's evaluation in almost a third of cases. In nearly half of the patients with RRMS with suspected SPMS, physicians felt that the report provided additional information as to whether the patients were in a transition phase from RRMS to SPMS. Of note, more than half of these cases were rated as not transitioned by inclusion of the quantitative report.

Recent research has shown that disability progression independent of relapse activity occurs early in the RRMS phase, probably involving the same pathophysiological mechanisms as in SPMS (Kappos et al., 2020; Kuhlmann et al., 2023; Tur et al., 2023). Consequently, a categorical and static distinction between relapsing and progressing forms of MS, as postulated by the Lublin classification, is increasingly questioned on scientific grounds (Giovannoni et al., 2022; Giovannoni et al., 2022). However, the Lublin classification also includes classifiers of inflammatory disease activity and of disability progression which better reflect the clinical reality and pathophysiology of the disease.

As per European Medicines Agency, available on‐label treatment options are determined by the categorization of MS as clinically isolated syndrome, RRMS, SPMS, or PPMS, as well as by disease activity. Availability of qMRI results may support the correct application of the Lublin classification, which in turn should inform treatment choices. As an important result of MAGNON, physicians interpreted the initial MRI report as indicating a change in therapy in 32.1% of patients with suspected transition to SPMS. However, an actual change in therapy was reported for only 16.7% of these patients at follow‐up. The reasons for this inconsistency are unknown. In particular, participating physicians were not surveyed regarding their confidence in the accuracy and validity of the qMRI reports or any contradiction to the routine MRI reports.

As the MAGNON project was conducted locally in Germany, the project population may not be representative of other countries. Owing to the noninterventional design, the visit schedule was not strictly enforced. Shorter or longer intervals between clinical appointments and MRI scans may have occurred according to clinical routine. Not all variables were collected for all patients and visits, leading to bias (skewing) in the results. Additionally, there is a possibility of an inclusion bias, although the study sites were expected to register all eligible patients who met the inclusion criteria for the project.

## 5 CONCLUSION

In conclusion, the MAGNON project confirms the feasibility of including qMRI reports, generated from routine MRI studies, in the routine clinical care of MS patients. qMRI data on lesion development and atrophy were regarded as useful by the treating physicians. In the task of applying the revised Lublin classification, the inclusion of the reports changed the characterization in particular for RRMS patients with suspected transition to SPMS. At present, the impact on clinical decisions appeared to be limited. The availability of qMRI is expected to increase, along with growing experience and confidence in the results.

## AUTHOR CONTRIBUTIONS

**Verena Isabell Leussink and Manda Jankovic**: Investigation; writing—review and editing. **Marie Groth**: Conceptualization; project administration; writing—review and editing. **Katrin Schuh and Inessa Schwab Sauerbeck**: Conceptualization; project administration; writing—review and editing. **Olaf Hoffmann**: Conceptualization; investigation; writing—original draft; data curation; writing—review and editing.

## CONFLICT OF INTEREST STATEMENT

Verena Isabell Leussink received research support, consulting fees and honoraria for lectures from Biogen, Novartis, Roche, and Teva. Manda Jankovic received research support and/or consulting fees and/or honoraria for lectures from Bristol Myers Squibb and Novartis. Marie Groth, Katrin Schuh, and Inessa Schwab Sauerbeck are employees of Novartis. Olaf Hoffmann reports speaker or consultancy honoraria and/or support for attending scientific meetings from Alexion, Bayer Healthcare, Biogen, Bristol Myers Squibb/Celgene, Janssen, Merck, Novartis, Roche, Sandoz, and Sanofi and research support from Biogen, Novartis, and Sanofi.

## FUNDING INFORMATION

Novartis Pharma Vertriebs GmbH, Germany

### PEER REVIEW

The peer review history for this article is available at https://publons.com/publon/10.1002/brb3.3548.

## Data Availability

The data that support the findings of this study are on file at Novartis Pharma GmbH and Novartis Pharma AG. Due to licensing restrictions, they are not publicly available. Data are, however, available from Inessa Schwab Sauerbeck (inessa.schwab_sauerbeck@novartis.com) upon reasonable request and may only be used with permission of Novartis Pharma GmbH and Novartis Pharma AG.
